# Sustainable Agriculture Solutions: Biodegradable Coatings for Enhanced-Efficiency Fertilizers Using Cellulose and Lignin

**DOI:** 10.1021/acs.jafc.5c01173

**Published:** 2025-05-20

**Authors:** Przemysław Boberski, Marek Główka, Kamila Torchała, Grzegorz Kulczycki, Nikodem Kuźnik

**Affiliations:** † Łukasiewicz Research Network, Institute of Heavy Organic Synthesis “Blachowania”, ul. Energetyków 9, 47-225 Kędzierzyn-Koźle, Poland; ‡ Faculty of Chemistry, 49569 Silesian University of Technology, ul. M. Strzody 9, 44-100 Gliwice, Poland; § Institute of Soil Science, Plant Nutrition and Environmental Protection, 56641 Wroclaw University of Life and Environmental Sciences, ul. Grunwaldzka 53 street, 50-375 Wroclaw, Poland

**Keywords:** fertilizers, slow-release fertilizers, controlled-release fertilizers, biodegradable coatings, modified cellulose, modified lignin

## Abstract

Current challenges in sustainable development and environmental protection are driving scientists and engineers to innovate and mitigate the negative impact of human activities on ecosystems. Strict legal requirements in the European Union (EU) underscore the need for changes to materials used in fertilizers because all components must be biodegradable by July 2024. The EU has published new guidelines for biodegradable materials based on ISO 17556, which assesses degradability in soil. One of the main directions to meet these expectations is the use of enhanced-efficiency fertilizers made from biodegradable materials. This review critically analyses recent advances in using natural raw materials as mineral fertilizer coatings, addressing a significant gap in the evaluation of their commercial viability. The properties of cellulose and lignin as coating materials and their modifications are discussed, including cellulose acetate, ethylcellulose, cellulose acetate butyrate, and phenolated and oxypropylated lignin. These findings provide practical guidance for fertilizer manufacturers seeking to comply with upcoming regulations while maintaining product efficacy.

## Graphical abstract

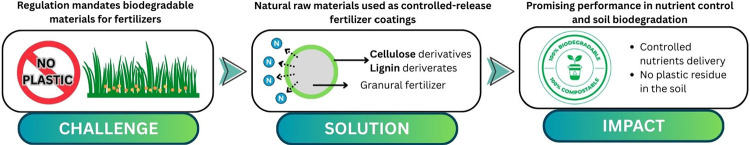

## 1 Introduction

In the face of the present challenges to sustainable development and environmental protection, there is an increasing demand for novel solutions from the scientific and engineering communities to mitigate the adverse effects of human activity upon the environment.^1,2,3,4^ Moreover, the stringent legal requirements that have been imposed on growers within the European Union serve to underscore the necessity for changes to be made in terms of the materials that are utilized.^5,6,7^ The main reason is the combination of a growing population with increased life expectancy. This implies a need to increase grain production by approximately 70% by 2050.^8^ Consequently, there will be an increase in demand for water, fertilizers, and pesticides. However, it is important to note that volatilization and leaching processes result in a loss of approximately 50 to 70% of fertilizers. This requires an increase in fertilizer utilization. The excessive use of these chemicals has been linked to a number of environmental concerns, including the eutrophication of lakes and rivers and the contamination of groundwater. In addition, it can result in significant economic losses.^9^ Furthermore, the use of chemical fertilizers impacts greenhouse gas emissions in the agricultural sector. This occurs because N_2_O is released during the conversion of a portion of nitrogen into nitrates when nitrogen fertilizers are applied to the soil.^10^ Furthermore, the global warming potential (GWP) of N_2_O exceeds that of CO_2_ by a factor of approximately 310.^11^ Additionally, CO_2_ and CH_4_ emissions are also closely associated with the use of chemical nitrogen fertilizers in agricultural systems.^12^ Therefore, improving fertilization efficiency and enhancing the ecological performance of fertilizers is essential. An effective approach is the use of coated fertilizers made from natural materials such as cellulose and lignin. Thanks to their wide availability and abundance, these biopolymers offer a promising path toward the sustainable production of valuable chemicals. Globally, lignin is produced at in annual volume of approximately 100 million tons, with an estimated market value of around USD 732.7 million.^13^ Lignin, an abundant and cost-effective natural polymer, has been the subject of extensive research aimed at its valorization. Over recent decades, its role has undergone a significant transformation: from a low-value byproduct primarily utilized as fuel or animal feed to a valuable precursor for the synthesis of high-value products. Similarly, cellulose, due to its renewable, biodegradable, and biocompatible nature, represents a highly promising resource for the development of environmentally sustainable materials in a broad spectrum of applications.^14^ In particular, the chemical conversion of plant-derived waste into coating materials for fertilizers presents considerable potential, offering a promising avenue for the development of sustainable agricultural technologies.

The persistent accumulation of nonbiodegradable polymers in agricultural soils presents a serious environmental challenge with significant implications for public health. Synthetic polymers, such as polystyrene, polyolefins, and polyurethanes, exhibit high resistance to degradation, allowing them to remain in the soil for prolonged periods. Their presence can lead to a progressive decline in soil quality, disrupt plant development, and negatively affect soil microbial communities. Moreover, these contaminants have the potential to enter the food chain through the uptake and consumption of contaminated agricultural products, thereby posing risks to both human and animal health.^15–17^ Therefore, it is imperative to promote sustainable agricultural practices and to identify alternative, environmentally benign packaging materials and agronomic solutions aimed at mitigating the adverse effects associated with the accumulation of nonbiodegradable polymers in the soil. Currently, the majority of coated fertilizers are formulated using nonbiodegradable polymers, which contribute to the increasing presence of microplastics in agricultural soils. Adopting biodegradable coating materials represents a promising strategy to address microplastic pollution and support the development of more sustainable soil management practices.^18^

According to the European Union Regulation on fertilizing products,^19^ all fertilizer components and their packaging must be made of biodegradable materials by July 2024. Currently, there are well-established methodologies for evaluating the biodegradability of materials. According to recent regulatory directives, coating materials used in fertilizers must demonstrate a minimum of 90% degradation in terms of organic carbon within 48 months after the complete release of active substances. These evaluations are guided by standardized protocols based on International Organization for Standardization (ISO) and ASTM methodologies. In the agricultural sector, there is increasing emphasis on the utilization of materials that not only fulfill their intended functions effectively, but also exhibit biodegradability. This paradigm shift supports waste reduction and contributes to minimizing the environmental footprint of agricultural practices.^20^

Enhanced-efficiency fertilizers (EEFs), made with biodegradable materials, ideally match this trend.^21^ Granulated fertilizers are excellent coating materials due to their physical form, durability and mechanical resistance.^22^

Granulated fertilizers have emerged as a promising choice for coating materials due to their distinctive physical characteristics, robust durability, and notable mechanical resistance. Coated fertilizers can be classified according to the coating material: organic and inorganic (Figure 1). Organic coatings may consist of resins, thermoplastic polymers, thermochemical or photocurable materials, or materials capable of transitioning to a vitreous state.^23^ Inorganic coatings include those based on sulfur and those based on naturally occurring minerals such as zeolite,^24^ gypsum,^25^ or hydroxyapatite.^26^ However, the addition of powdered coating materials, especially inorganic ones, can lead to significant dusting, which poses health and environmental concerns.^27,28^

**Figure 1:**
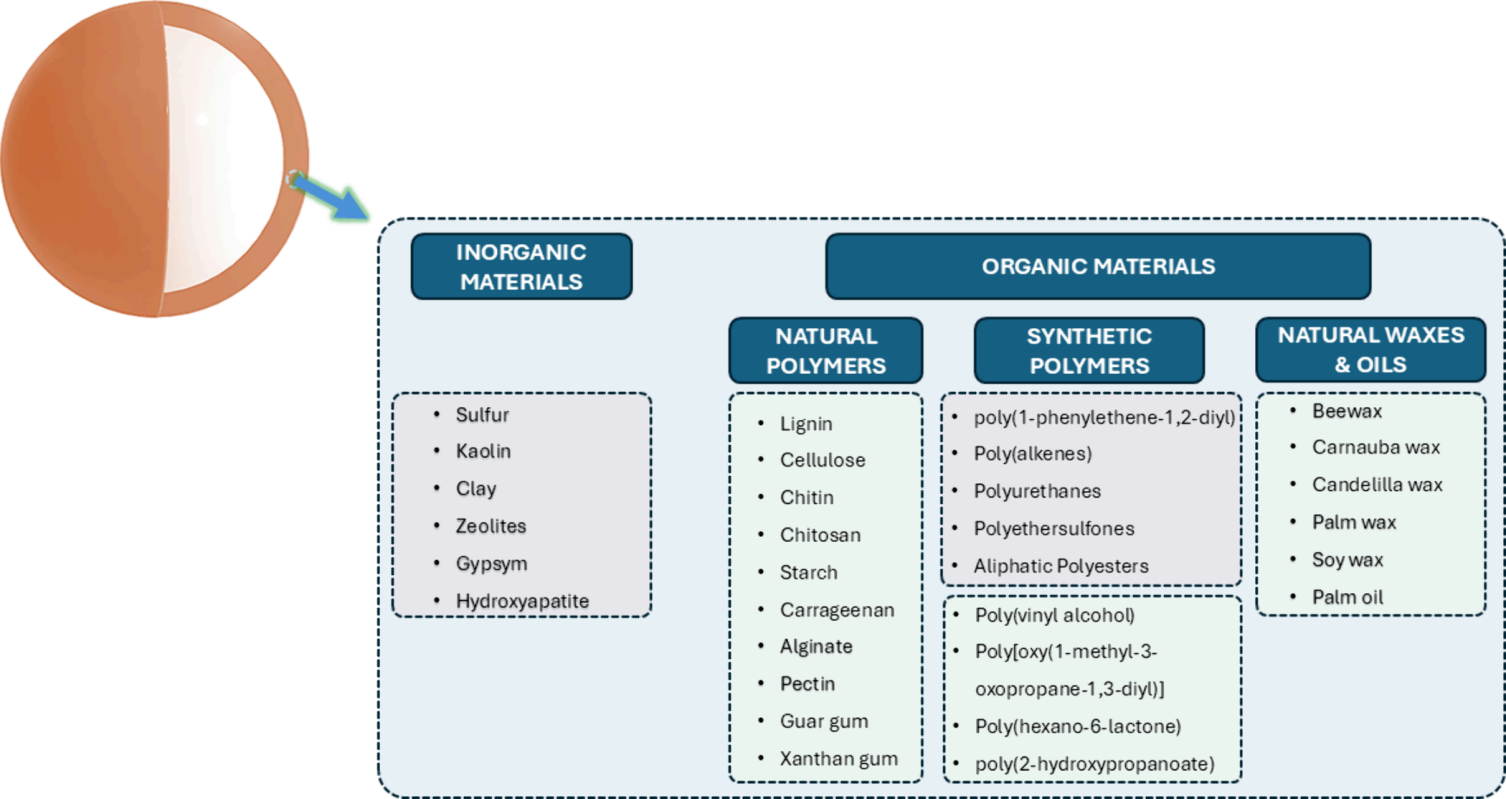
Materials used to coat granular fertilizers.

Modifications of natural polymers, such as lignin and cellulose found in plants, are emerging as promising raw materials for the production of shells for granulated fertilizers.^29^ The adoption of biodegradable materials in agriculture not only ensures effective protection and controlled release of nutrients, but also aligns with the overarching goals of sustainable development, which prioritize environmental stewardship and adherence to regulatory standards governing the use of biodegradable materials in agricultural practices. Biodegradable polymers are classified into two categories, synthetic and natural polymers - based on their origin.

However, coated fertilizers are not yet defined in formal terms. Existing standards, including those established by the European Union (EU), ISO, and GB (Chinese standards), categorize controlled-release fertilizers (CRFs) and EEFs under the broader classification of slow-release fertilizers (SRFs). However, authentic CRFs should be designed based on condition-responsive effects or rhizosphere priming effects, which are essential to meet the specific requirements of different plant species.^30^ Nevertheless, to date, none of the commercially available products or research results do not meet these requirements and are therefore often classified as SRFs.

The aim of this review article is to critically analyze research on the use of organic polymers—cellulose and lignin—as coating materials for fertilizers. The focus is on evaluating the proposed coating materials in terms of their commercialization potential. Based on the analysis of the literature, new trends were identified and perspectives for future research directions were outlined, with the aim of introducing lignin- and cellulose-based coatings into the industry. The review was carried out according to the 27 principles outlined in the Preferred Reporting Items for Systematic reviews and Meta-Analyses (PRISMA).^31^ The flow diagram is shown in the Supporting Information. Articles were selected using the Scopus and Web of Science databases using keywords fertilizer, lignin, cellulose, coated, and encapsulated.

## 2 Discussion

### 2.1 Purpose of Coating Nitrogen Fertilizer Granules

Nitrogen is an element that has a significant impact on plant yield because it plays an essential role in plants as an integral component of proteins, nucleic acids, chlorophyll, coenzymes, phytohormones, and secondary metabolites.^32^ Plants absorb nitrogen mainly in its ionic forms: nitrate (NO_3_^–^) and ammonium (NH_4_^+^) through their roots (Figure 2).^33^ Although the supply of nitrogen (N_2_) in the air is abundant, unfortunately, only plants capable of microbial symbiosis can take advantage of it.^34^ The second limiting factor in the availability of nitrogen for agricultural crops is its small and highly variable amount in mineral form in the soil.^35^ The above factors determine the necessity of introducing mineral nitrogen compounds in the form of fertilizers during the growing season of crops to meet their nutritional needs. Among nitrogen fertilizers, urea accounts for the largest share of global consumption (49.3%), with smaller amounts of nitrogen supplied to agriculture in the form of ammonium nitrate (5.6%) and ammonium sulfate (3.9%).^36^

**Figure 2:**
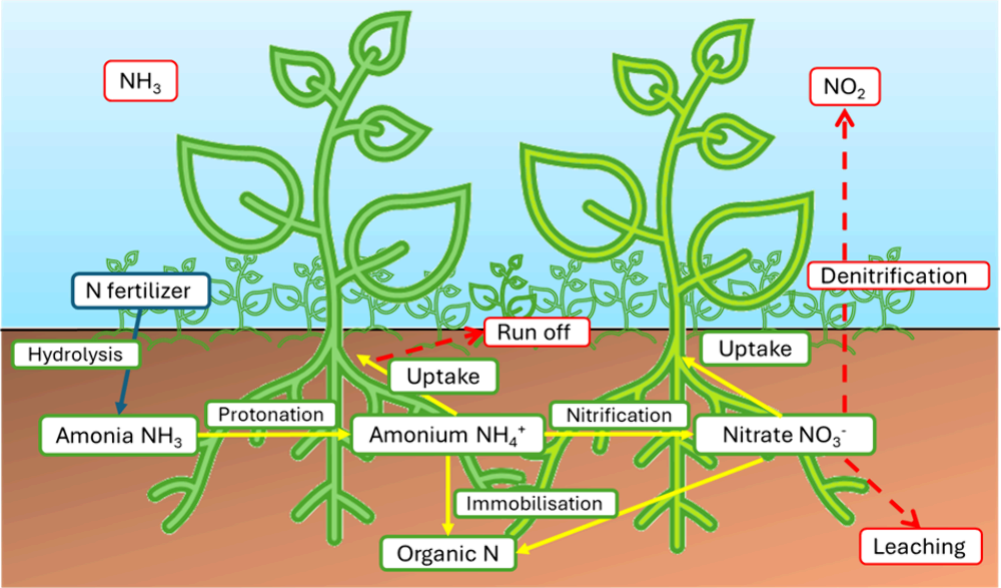
Illustration of the nitrogen cycle in the soil.

Once nitrogen is applied as fertilizers to the soil, it would be optimal if plants absorbed as much as possible. Unfortunately, the efficiency of nitrogen use (NUE) in agriculture is low, ranging from 30 to 60%.^37^ This is due to several factors, such as the conversion of nitrogen in soil to nitrate forms, which, if not taken up by plants, leach out and the failure to match the timing of fertilizer applications with the intensity of crop vegetation, and improper estimation of plant nutrient needs.^38^ The purpose of coating the nitrogen fertilizer granules is to slow down the release of available forms of nitrogen, that is, to align the availability of nitrogen to plants according to the intensity rate of intensity of their vegetation.^39^ The term “controlled-release fertilizer” accurately describes fertilizers where the rate, pattern and duration of nutrient release are well understood and can be managed during CRF production. In contrast, “slow-release fertilizers” release nutrients more gradually than usual, but the rate, pattern, and duration of this release are not as precisely controlled. Generally, both terms could be used synonymously.^40^ For the consistency of the text, both the CRF and the SRF will be referred to as the EEF. Although the terms *encapsulation* and *coating* are often used interchangeably in the context of the EEF issue, it is important to clarify the distinction between them for the sake of textual coherence. *Encapsulation* refers specifically to the process in which nutrients are enclosed within an external medium, which also serves as the basis for the controlled release mechanism. In the context of fertilizers, this material may undergo chemical or physical modifications to facilitate the controlled release of nutrients contained within its matrix. The coating process is defined as the application of a material to the surface of granular fertilizers. This study exclusively encompasses research that involves the application of coating processes.

### 2.2 Cellulose: Structure, Properties, and Industrial Applications

Cellulose is a natural polymer that has been utilized by humans for hundreds of years. It is a common component of wood along with lignin and hemicellulose. Initially, it was used for the production of tools to construct buildings, bridges, and ships.^41^

Cellulose is a renewable polymer that is widely available in nature, particularly in plant fibers, where it constitutes approximately 40% of the carbon fraction. It plays a crucial role as a structural element in the complex architecture of cell walls in plants, animals, algae, fungi, and minerals. Its abundance and versatility make it an excellent choice for a wide range of applications. Cellulose commonly occurs, usually accompanied by hemicelluloses, lignin, and small amounts of extractive substances. Wood contains approximately 40–50% cellulose. Similarly, cellulose is present within sugar cane bagasse (35–45%), bamboo (40–55%), straw (40–50%), flax (70–80%), hemp (75–80%), and jute (60–65%). Cotton is a highly pure source of cellulose, containing more than 90%.^42,43^ Bacteria from various genera, including *Gluconacetobacter*, *Agrobacterium*, *Pseudomonas*, *Rhizobium*, and *Sarcina*, have the ability to produce bacterial cellulose from glucose and other carbon sources.^44^ The resulting product is a fibrous network that is free from lignin, pectin, hemicelluloses, or other biogenic products. This bacterial cellulose is highly crystalline and has a high degree of polymerization.

In 1837, the French chemist Anselme Payen started researching and eventually discovered cellulose. Payen showed that when various plant materials were treated with ammonia, they produced fibrous substances. The process involved treatment and extraction with water, alcohol, and ether. The material obtained was named “cellulose’ (from the Latin *cellula* or “little cell”) by the French Academy. Cellulose is a structural polysaccharide found in plant cells. It is made up of d-glucose units linked by β-1,4-glycosidic bonds, forming a linear polymer. In simple terms, cellulose is a type of sugar that gives plants their structure. It consists of two glucose molecules and could also be considered a polyacetal (syndiotactic) of glucose. The cellulose chain consists of repeating cellobiose units, each consisting of two glucose units in the chair conformation, rotated 180° relative to each other (Figure 3). The general structure can be expressed by the formula (C_6_H_10_O_5_)_*n*_, where *n* represents the number of glucose units.^45^

**Figure 3:**
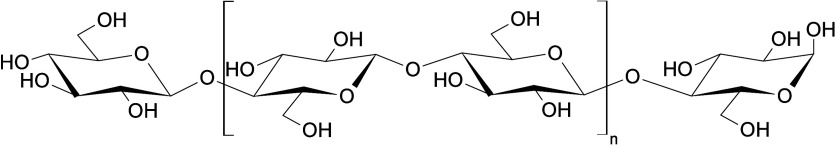
Schematic of the molecular structure of a cellulose biopolymer.

Depending on the plant species, its habitat, and external factors such as fertilization or weather conditions, the number of glucose units may vary. Cellulose exists as a polymer with varying molecular weights, typically ranging from 160.000 to 560.000 g/mol.^46^ The macromolecules of cellulose arrange themselves into specific chains, which are not as perfect as crystals, but are somewhat ordered. The degree of organization depends on the proportion of crystalline and amorphous regions, and there are no clear boundaries between them. Native cellulose typically has a degree of crystallinity of around 70%. However, it is important to note that this parameter varies depending on the cellulose source. For instance, cotton has a much higher degree of crystallinity than wood.^47^

Every year, a significant amount of cellulose is produced from a variety of sources. According to a report by Data Bridge Market Research, the global cellulose market is projected to experience robust growth. According to recent market research, the value of the market is expected to increase from $144.21 billion in 2024 to $226.79 billion in 2035, with an impressive annual growth rate of 4.20% anticipated during the period from 2024 to 2035.^48^ Cellulose, in its various forms of acetates, ethers, and esters, is a vital component in several sectors, such as pharmaceuticals, food, textiles and cosmetics, where it serves as a crucial ingredient for thickening, stabilizing, emulsifying, and membrane-forming purposes. The natural sources segment is set to dominate the global cellulose market because of the sustainable and renewable nature of cellulose, making it a preferred choice for environmentally conscious industries and consumers. In particular, the Asia-Pacific region is expected to lead the global cellulose market, particularly in the textile and pharmaceutical industries, which are the key consumers of cellulose products. Between 2023 and 2030, Europe is poised for dynamic growth, driven by strict regulations on product quality and sustainable development. As a result, there will be a significant demand for high-quality cellulose materials in this region.^49^

Cellulose is a hygroscopic substance that absorbs approximately 8–14% of water at 20 °C and 60% humidity.^50,51^ Although cellulose swells when it absorbs water, it does not dissolve in it. Cellulose exhibits insolubility in diluted acid solutions; however, in concentrated acids, partial chain degradation can occur, allowing for dissolution. In an alkaline environment, cellulose swells and partially dissolves, primarily affecting components with low polymerization. It is noteworthy that cellulose does not melt, but instead undergoes decomposition as temperature increases. Decomposition occurs above 180 °C and complete destruction through carbonization occurs at temperatures ranging from 250 to 300 °C.^52^

#### 2.2.1 Chemically Modified Cellulose

Pure cellulose without any modification (chemical, physical, or biological) is not ideal as a coating material in CRFs due to its physicochemical properties. However, by functionalizing it, properties such as solubility, mechanical strength, or film-forming ability can be modified. The reactive hydroxyl groups allow for relatively simple modification through various techniques, e.g. esterification, etherification, grafting modification (utilizing monomers such as acrylonitrile or acrylamide), oxidation modification, and cross-linking modification.^53^ Esterification and etherification reactions are two commonly used modifications, products of their use in coated fertilizer these modifications have been comprehensively described below in section 2.2.2.

Micro- and nanocellulose (CMF/CNF) are extensively used as components of biodegradable EEFs, aiding in the controlled release of nutrients and enhancing their efficiency.^54^ Research indicates that cellulose nanofibers can significantly modify the properties of polymer matrices, allowing precise adjustment of nutrient release rates (e.g., KNO_3_) in both aqueous and soil environments.^55^ Furthermore, cellulose nanocrystals, when used in combination with other polymers such as poly­(vinyl alcohol), have been shown to extend nutrient release in the soil for up to 30 days.^56^ The central theme of this study is research on fertilizer coatings materials, and therefore the use of CMF and CNF is not discussed further.

Cellulose can be esterified by reacting with a nitration mixture, which typically consists of concentrated nitric acid and concentrated sulfuric acid, or another binding agent that forms water in the reaction, such as *o*-phosphoric acid. The term “nitrocellulose” may be misleading because it refers to the ester of cellulose and nitric acid, rather than the introduction of a nitro group (Figure 4). Nitrocellulose is classified as both an explosive propellant and an explosive disintegrant. It is important to note that commercial products are a mixture of compounds with varying degrees of substitution (DSs), ranging from unreacted cellulose to products with complete substitution of hydroxyl groups.^57^ Mentioned above modification of cellulose has not been employed in the production of fertilizers as a coating material.

**Figure 4:**
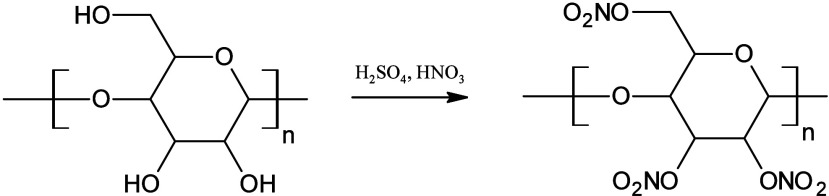
Reaction scheme for obtaining nitrocellulose.

Cellulose esterification is a reaction that takes place in a homogeneous system that requires the dissolution of cellulose. The most commonly used acid for esterification is concentrated acetic acid, while the esterifying agent can vary depending on the desired ester. Carboxylic acids, acid chlorides, or anhydrides can be used for this purpose. Anhydrides offer several advantages, one of which is the elimination of low-molecular-weight byproducts.^58,59^ This is especially beneficial when working with carboxylic acids because the byproduct is water.

Figure 5 illustrates the cellulose esterification reaction using anhydride, which can produce a variety of materials depending on the agent used. The acylation process is conducted under acidic conditions with sulfuric acid as a catalyst, involving a reaction with acid anhydrides. The resulting product is then isolated through precipitation by introducing the homogeneous reaction mixture into water. Subsequently, the solid product is separated, washed to attain a neutral pH, and dried.^60^ The esterification of cellulose results in the formation of products that are capable of dissolution in conventional organic solvents. This makes them suitable for utilization as a coating agent in the context of spray coating method.^61,62^

**Figure 5:**
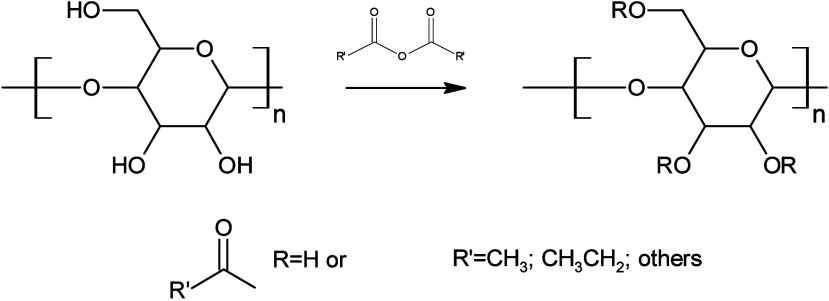
Reaction scheme for cellulose esterification using anhydride.

Cellulose acetate (CA) is the most widely used cellulose ester. The global CA market is growing steadily. It reached 2.0 million tons in 2022 and is projected to reach 2.4 million tons by 2028, with a compound annual growth rate (CAGR) of 3.6% throughout 2023–2028.^63^

The industrial method for producing CA involves activating the cellulose by immersing it in a solution of acetic acid in the presence of a catalyst. Subsequently, acetic anhydride was introduced into the reaction environment to initiate functionalization. Temperature control is crucial to this process because it is highly exothermic. This results in the production of cellulose triacetate, which is soluble in a given environment. Acetic acid is added to the mixture to stop the reaction; the acetic acid reacts with the remaining unreacted acetic anhydride. Sulfuric acid serves as an excellent catalyst in the hydrolysis stage, leading to deacetylation and results in a product with a DS typically in the range of 2.4–2.7, usually less than 3. The cellulose diacetate obtained from this process precipitates in water as a white substance that possesses excellent scratch resistance, insulating properties, and antistatic properties. Additionally, it is highly resistant to oils and fats.^64^

The properties of CA are mainly dependent on the DS and the molecular weight. In general, CA is a versatile material with a range of properties that can be tailored to specific needs. The hydrophobicity of CA decreases as the number of ester substituents and the length of their chain increases. The mechanical and barrier properties of coating materials are significantly impacted by their affinity for water. Notably, cellulose fibers have a moisture content of approximately 12%, while diacetate fibers have 6.5%, and triacetate fibers have 3.2%.^65,66^ The solubility of CA depends on its DS. CA with a DS of 2–2.5 dissolves in acetone, dioxane, and methyl acetate. For higher acetylated concentrations, dichloromethane is the preferred solvent. CA with a lower DS is the preferred option from an application perspective because of the negative environmental impact of solvents like dichloromethane. CA has a wide range of applications, depending on the acetic acid content. For example, acetate, with approximately 55% acid content, is primarily used for the production of artificial fibers, while the 52–53% acid content is suitable for lacquers and plastics. Acetate with 58–61% acid content is used to produce films and foils.^67^

The etherification of cellulose yields materials classified into simple ethers, mixed ethers, and hydroxyethers. Cellulose ethers are produced by reacting halides or sulfates of suitable alkyls or alkylaryls with alkali cellulose. High-quality cellulose ethers with consistent properties can be obtained. Alkali cellulose refers to cellulose treated with a 16–18% sodium hydroxide solution. The properties of alkali cellulose and the cellulose ether production process are influenced by several key factors, including the concentration of NaOH, the process duration, and the temperature.^47^

Cellulose ethers have wide-ranging applications, serving as lacquers, binding agents, emulsifiers, insulating coatings, and protective colloids. These derivatives introduce new properties such as increased hydrophilicity, resistance to microbial action, improved chemical and thermal resistance, and enhanced mechanical properties. Methyl- and ethylcellulose are among the most popular cellulose ethers due to their simple ether structure.^68^ Cellulose ethers have been found to be a satisfactory coating agent due to their nontoxicity and good mechanical properties. The modified solubility of cellulose ethers makes them suitable for coating processes through various coating methods.

#### 2.2.2 Modified Cellulose: EEFs

Cellulose is a widely available and biodegradable material with significant potential to coat granulated fertilizers. Its use in this application could lead to advancements in the fertilizer industry. However, its highly hygroscopic nature may cause swelling upon contact with water, potentially disrupting its continuous structure. This challenge can be addressed by modifying cellulose into a more processing-friendly form. Additionally, while cellulose is insoluble in many solvents, limiting its direct applicability in solvent-based processes, various methods exist to enhance its solubility and other properties. For instance, functionalization reactions such as esterification or etherification can be conducted, as described in section 2.4. Additionally, the combination of cellulose with other materials to create two- and multilayer compositions can enhance its performance even further. Cellulose-based fertilizers can be classified into two main categories: cellulose-coated fertilizers, such as granules coated with ethylcellulose (EC) or hydroxypropylmethylcellulose (HPMC), which slow the release of nitrogen, phosphorus and potassium; chemically modified fertilizers, where cellulose contains amino, carboxyl, or sulfonic groups that improve nutrient retention.^69,70^

A publication by Qiao et al.^71^ introduced a urea-based fertilizer with a double coating system, designed to ensure a high-quality fertilizer that should yield excellent results. The coating system comprised two layers: an inner layer of EC and an outer layer made of starch, another natural polymer. To ensure the highest quality, the fertilizer particles were meticulously sieved to obtain a diameter of 2–3 mm and particles with surface defects were removed. For the coating process, a solution of EC and stearic acid (in a ratio of 9:1 w/w) dissolved in ethanol was used at a concentration of 6.25% (w/v). The first coating layer was applied by pressure spraying the urea granules with an EC solution. Subsequently, a modified starch was applied, displaying superabsorbent polymer (SAP) capabilities. Modified potato starch exhibited a water absorption capacity of 110 g/g. The release behaviors were assessed under soil conditions. When comparing the nutrient release profiles, the fertilizer coated only with EC released 100% of its nutrients in 48 h; on the contrary, the fertilizer with an additional starch layer released all nutrients in 96 h. When considering the amount of coating applied to the fertilizer, it is important to note that, for a single layer (EC), the coating constituted 10% by weight relative to the core fertilizer. However, for two layers, the coating proportion increased to 45%, with the inner layer constituting 10% and the outer layer 35% by weight.

Although the study by Qiao et al. provides insights into using double-layer coatings for urea-based fertilizers, significant limitations hinder its practical application. The excessive proportions of coatings, modest slow-release improvements, solvent use, potential mechanical instability, environmental concerns, and manufacturing complexities all point to challenges that must be addressed before this approach can be considered commercially viable or environmentally sustainable. More research is needed to refine the coating process, improve the long-term release of nutrients, and evaluate the environmental impact in a comprehensive way.

An intriguing application of modified cellulose in coated fertilizers is the concept described by Milene M. Costa’s team.^72^ Their team developed fertilizers coated with ethyl cellulose, polyhydroxybutyrate, or both, without distinguishing the boundary between phases. Furthermore, they conducted a thorough study of the effects of various additives, including Triton X-100, cetyltrimethylammonium bromide (CTAB) and sodium lauryl sulfate (SLS), on the coating process. The polymers were applied using the spray method, with acetone and chloroform as the solvents. Two coating methods were employed: immersion and spraying. In the immersion method, the fertilizer was submerged in the coating mixture and then the solvent was allowed to evaporate. For spraying, a spray nozzle was used to deliver the polymer onto the surface of the granule. After coating, the fertilizers were tested for nutrient release in a distilled water test. The study shows that of coating the fertilizer reduces nutrient release, but the reduction is not enough for agricultural use. The authors concluded that the unsatisfactory results were due to the low amount of coating applied, which did not exceed 2% of the weight of the core fertilizer in any of the prepared samples. While the concept of using modified cellulose in coated fertilizers is innovative, the research by Costa et al. suffers from significant limitations, including the low coating percentage, reliance on toxic solvents, use of environmentally harmful surfactants, and lack of clarity regarding polymer phase boundaries. Furthermore, the study’s testing methods do not simulate real-world agricultural conditions and its results are ultimately insufficient to meet the demand for SRFs. To make this approach viable, substantial improvements in the coating process, material selection, and testing protocols are necessary.

The work of Ni et al.^73^ also utilized the concept of double coating. Initially, the granule was prepared with a sodium alginate matrix, followed by a coating with an inner layer of EC and an outer layer of cross-linked poly­(acrylic acid-*co*-acrylamide) (P­(AA-*co*-AM)). To produce the multifunctional fertilizer, the granules of fertilizer were immersed in a polymer solution to apply a superabsorbent outer coating. This coating slows the release of nutrients and allows for water absorption. The effectiveness of the fertilizers was performed through a soil test. After 30 days of incubation in the percentage of soil, the nutrient release was over 75%. In conclusion, while the double coating system explored in this research offers some advantages in water absorption and nutrient release control, it falls short of delivering the slow-release capabilities expected of modern fertilizers. The study lacks long-term data, raises environmental concerns, and presents challenges in large-scale application. Further research should focus on improving the release profile, improving the environmental sustainability of the materials used, and ensuring the practicality of the coating process.

Lubkowski et al.^74^ have developed a fertilizer coating made entirely of EC. They conducted a series of experiments in which a multinutrient fertilizer (containing nitrogen, phosphorus, and potassium) was coated with varying amounts of EC (ranging from 0.165 to 0.285 of polymer in relation to the fertilizer core). The coating was produced by immersing the fertilizer in a 10% EC solution in ethanol. By repeating the granule coating procedure multiple times, fertilizers with varying degrees of coating were prepared. Mechanical strength tests and nutrient release profiles recorded in the water test were used to evaluate the fertilizer samples. The results demonstrated that the coated fertilizer exhibited five times greater durability than the uncoated counterpart. Furthermore, on the water nutrient release test, it can be concluded that the fertilizer meets the standards for coated fertilizers. It released no more than 75% of its nutrients in a 28-day period, but only when the polymer-to-fertilizer ratio is higher than 0.21. In conclusion, while Lubkowski et al. (2019) demonstrate the potential of EC as a fertilizer coating, several key limitations exist. The study lacks real-world soil testing, relies on a high polymer-to-fertilizer ratio, and raises questions about the environmental impact of EC coatings. Additionally, the approach may be cost-prohibitive for widespread adoption and may not be versatile enough to accommodate various agricultural needs. More research is needed to refine the coating method, evaluate long-term environmental effects, and ensure the practicality in diverse farming systems.

An interesting approach is presented in Yuan’s work, where he and his team developed EEF from start to finish, i.e. from the extraction of rice straw and then the modification of cellulose by etherification. Subsequently, they synthesized the modified core by mixing urea with clay and then applied ethyl cellulose as a coating from the ethanol solution by means of spray application.

The prepared fertilizer was assessed both in deionized water and in soil conditions with the use of wheat. An aqueous test was conducted to determine the coating relationship between the concentration and the rate of release of the ingredients. The findings demonstrated that as the concentration of the envelope increased, the rate of release of the ingredients decreased. The study revealed that the use of an uncoated fertilizer on the first day resulted in the release of more than 85% of nutrients. On the contrary, the fertilizer containing 5% of the coating discharged approximately 64% of the nutrients, while the fertilizer comprising 15% of the coatings by weight released 32.31% of the nutrients. Based on soil tests, the application of EC coated urea has been demonstrated to enhance nitrogen management in the soil–plant system compared to conventional urea. Treatments demonstrated a delayed and sustained nitrogen release, which is more in accordance with crop uptake, thus reducing leaching losses by up to 70%. The coating not only controlled the release of nutrients, but also preserved the integrity of the fertilizer and facilitated efficient root nutrient absorption. However, the results of the biodegradability test were not disclosed; based on a review of scientific literature, the authors of the study that EC is a fully biodegradable material.^69^

In the above examples, the utilization of ether derivatives of cellulose was demonstrated. It is noteworthy that materials from the group of ester derivatives are also of general interest. CA, the simplest derivative and widely recognized material for membranes, has been utilized effectively as a coating substance. A fertilizer prepared using CA was described by Jarosiewicz and Tomaszewska’s^75^ work. The purpose of the outlined works was to compare the effectiveness of CA, polysulfone (PSF), and polyacrylonitrile (PAN) as coating materials for granular fertilizers. The coatings using the phase-inversion method with DMF and acetone as solvents were prepared. The publication provided detailed information on the morphology of the prepared fertilizers and the results of the water nutrient release test. In a comparison of CA to fertilizers with PAN and PSF fertilizers, it was discovered that it released nutrients at the fastest rate. It should be noted that CA fertilizer was the only one among them that contained biodegradable material. The fertilizer with the CA coating released its components rapidly, with 66.5% of P_2_O_5_, 49.6% of K^+^ and 35% of NH_4_^+^ being released after 5 h of testing.^75^ In conclusion, while the study by Tomaszewska and Jarosiewicz offers valuable insights into the use of CA as a coating material for fertilizers, it suffers from significant limitations. The rapid nutrient release profile, the reliance on harmful solvents, and the lack of comprehensive testing under realistic agricultural conditions limit the practical applicability of the findings. Additionally, the study’s narrow focus on nutrient release and the absence of long-term environmental impact assessments call into question the broader utility of CA-coated fertilizers in sustainable agriculture. Further research is needed to refine the coating process, test under soil conditions, and explore safer and more sustainable solvent options.

Other work described by El Assimi et al.^76^ explains how a coated fertilizer was prepared using granulated diammonium phosphate as the core of the composition. The authors opted for a blend of CA and lactic acid polymer for the coating, applied via the spray method. Overall, this approach proved to be effective in achieving a well-coated fertilizer granule. The solvent mixture of THF: acetone (1:1) was used for the coating process. To ensure an even distribution of the coating solution on the surface of the fertilizer granule, the process was carried out in a rotating coating drum. The results demonstrate the effectiveness of the fertilizer production process. A wide range of fertilizers, with different coating materials, including polymer blend coatings (4:1, 1:1, 1:4) as well as pure CA and PLA, was prepared. To assess the release of nutrients, a standard water test was conducted. The fertilizer with a PLA:CA ratio of 4:1 was the most effective, releasing 24.5% of potassium after 24 h and the entire nutrient content within 46 h. The results do not provide conclusive evidence to confirm that the fertilizer obtained belongs to the slow-release category. However, it is important to note that in the best-case scenario, the coating only represented 3.7% of the total weight compared to the fertilizer core. It is theorized that increasing the proportion of the coating may help reduce the rate of nutrient release. Furthermore, El Assimi et al.^76^ conducted another experiment utilizing black liquor and CA as coating materials for fertilizers, resulting in two series of fertilizers with varying amounts of coating. The core of the fertilizer consisted of granulated ammonium phosphate. The spray method was used to produce fertilizers, where the black liquor was sprayed with water and the CA was sprayed from an acetone solution. Fertilizers with 0.5%, 1%, and 2% coating by weight relative to the fertilizer core were prepared. Samples coated with black liquor released nitrogen evenly, but for a significantly shorter period than samples coated with CA. The fertilizers were evaluated in a 96 h water test. For CA-coated samples, nutrients were fully released after approximately 96 h. On the contrary, the samples coated with black liquor showed complete nutrient release after about 80 h.

While El Assimi et al. (2021) present a novel approach to fertilizer coating using biodegradable polymers, the research suffers from several key limitations. Rapid nutrient release profiles, insufficient coating thickness, and reliance on harmful solvents raise concerns about the practical applicability and sustainability of proposed fertilizers. Furthermore, the use of standard water tests as the sole evaluation method, and the lack of mechanical property testing. Future research should address these shortcomings by testing fertilizers in soil environments, exploring safer solvents, and evaluating the economic feasibility and environmental impact of biodegradable coatings.

In other work, the use of various polymeric materials, such as sodium alginate, EC, and CA, along with inorganic coatings, was explored. The core of the fertilizer consisted of granulated biochar saturated with nitrogen by impregnation with urea in a periodic reactor. To evaluate the effectiveness of the fertilizers, experiments were conducted using soil columns with plants. In this study, the amount of nitrogen, including nitrates, nitrites, ammonium, and urea, was assessed as they passed through the column. Coatings were prepared using the phase-inversion method. The experiments revealed that the use of each coating significantly slows the nutrient release rate. Based on CA-coated results, the fertilizer released nitrogen for the longest time, resulting in the lowest release rate of microelements.^77^ Although the research by González et al. (2015) contributes to the growing body of literature on CRFs, it is hindered by several limitations. The narrow focus on nitrogen release, the lack of field testing, and insufficient discussion of the interactions between biochar and coatings restrict the relevance of the findings. The use of phase inversion coating methods, in conjunction with the absence of economic and environmental impact analysis, gives rise to concerns regarding the sustainability and feasibility of the proposed solutions. Moreover, the limited exploration of microelement release, variability in coating performance, and short nutrient release assessment period diminish its contribution to a holistic understanding of SRF technology. Addressing these shortcomings in future research would help advance the development of more effective, scalable, and environmentally friendly fertilizer solutions.

Ester derivatives are frequently used in the composition of coated fertilizers in multicomponent systems, similar to the use of ether derivatives. Wu and Liu^78^ prepared a two-layer fertilizer. The internal layer was composed of CA, while the external layer was made from a composite with superabsorbent properties (poly­(acrylic acid-*co*-acrylamide)/nonexpanded vermiculite). The research suggests that the prepared fertilizer has slow-release properties and is capable of water storage. The outer layer has a remarkable ability to absorb water up to 72 times its own mass. The internal coating, comprising CA, was prepared using the phase-inversion method. This layer was applied expertly from an 18% acetone solution. The evaluation of the fertilizer was carried out through a soil test using spectrophotometric and elemental analysis to determine its nutrient components. The results were promising, indicating that the prepared fertilizer released no more than 75% of its nutrients during a one-month incubation test. However, further tests, particularly a water test is required to definitively classify it as a SRF. The use of this unique coating material system not only decelerates the release rate of macro- and microelements, but also ensures a more efficient and effective fertilization process. Wu and Liu (2008) research on a dual-layer coated fertilizer offers an intriguing approach that combines slow nutrient release with water retention capabilities. However, the study is hindered by several limitations, including the lack of a long-term nutrient release analysis, concerns over the use of environmentally harmful solvents, and the absence of a water test to confirm slow-release properties. Additionally, the complexity of the two-layer system may pose challenges for large-scale production, and potential interactions between the coating layers could affect the fertilizer’s performance. To address these shortcomings, future research should focus on conducting long-term field tests, exploring environmentally friendly coating methods, and comparing the dual-layer system with other established slow-release technologies. Addressing these issues would provide a more comprehensive understanding of the viability for commercial agricultural use.

In Wang’s publication,^79^ other ester derivatives, such as cellulose acetate butyrate (CAB), have also been used successfully in the formulation of coated fertilizers as an internal layer of the coating. The fertilizers were coated on a rotating granulation disc using a composite with superabsorbent properties based on modified chitosan, poly­(acrylic acid) , and attapulgite clay. The core of the fertilizer was synthesized using ammonium zinc phosphate, while the internal layer was made of CAB and sprayed using an ethyl acetate solution. Finally, the external layer was obtained by incorporating a ground CMCS-*g*-PAA/APT composite into the system. The results suggest that the use of a single CAB layer can significantly reduce nitrogen release. The water tests showed that the uncoated fertilizer released 98.5% nitrogen after 24 h, whereas utilizing a single CAB layer reduced this value to 15.6% within the same time frame. A study comparing the effect of the number of layers on the nitrogen release profile for the fertilizer coated with a single layer and triple layers was also conducted. During the 30-day long-term test, the fertilizer with a thicker coating released 15.6%, 70.3% and 87.5% after 1, 15, and 30 day(s) respectively, while the fertilizer with a thinner coating released 28.3%, 85.3%, and 96.5% after 1, 15, and 30 day(s) respectively. The use of the composite as an additional outer layer allowed the fertilizer samples to meet the requirements of SRFs. In the 28-day test, 69.1% of the fertilizer was released and an increase in the fertilizer’s water sorption of the fertilizer was observed, improving its ability to retain water in the soil. Although the research highlights significant reductions in nitrogen release and improved water retention, several limitations are apparent. The multilayer coating system increases production complexity and costs, potentially limiting scalability. The use of volatile organic solvents, such as ethyl acetate, raises safety and environmental concerns, with no sustainable alternatives suggested. The 30-day evaluation period is insufficient to assess long-term performance or suitability for a full growing season. The lack of field trials under real-world conditions reduces the practical applicability of the results. More research is needed to address its economic, environmental, and practical challenges, including sustainable material use, long-term testing, and field trials.

In another study, Lu et al.^80^ explored the utilization of another cellulose ester derivative, commercially available cellulose acetate phthalate (CAP), in the composition of a coated fertilizer as the inner layer of the coating. Externally, a composite with superabsorbent properties based on modified chitosan, poly­(acrylic acid) , and attapulgite clay was employed. The fertilizer was applied to a rotating granulation disc. The core of the fertilizer was synthesized ammonium zinc phosphate. The primary goal of the described experiment was to establish diffusion models and kinetic equations for nitrogen release and its modeling. However, when considering how the amount of coating applied affected the rate of nitrogen release, it could be concluded that doubling the amount of coating material allowed for a 2-fold increase in the nitrogen release in water test. The study lacks a real-world evaluation of the agronomic performance, does not address the environmental impact of CAP and synthetic polymers, and omits long-term testing to assess the durability and performance. Additionally, the potential impact of the superabsorbent composite’s water retention on coating integrity and nutrient release under wet conditions remains unexplored.

The utilization of varied forms of cellulose and their ability to form hydrogel materials is described in the work of Kassem and his team, who prepared an experiment in which cellulose derivatives and regenerated cellulose were used to create an encapsulating material with hydrogel properties. The processes involved the use of granulated monoammonium phosphate, which was encapsulated through the implementation of a spray method. The prepared EEF was evaluated under both water and soil conditions. Two thicknesses of coating were applied, 27 and 71 μm. The thicker layer was found to allow a slower rate of nutrient release than the thinner layer. The experiment revealed that the application of the thicker coating resulted in the release of 58% of the fertilizer during the initial hour of the water test, with complete release of nutrients occurring after 12 h. Under soil condition, the same sample was 100% of nutrient after 18th day and about 58% nutrients after first day of the test. The cross-linking condition and thickness were subjected to rigorous evaluation. It was established that a higher cross-linking density leads to slower diffusion and lower release rates because of the reduced free volume and pore size in the hydrogel network. In Kassem’s study, the biodegradability of the material was determined by measuring the loss of dry coating material in the soil. The test was carried out over a 24-day period, during which a weight loss of 63.15% and 74.38% was observed in two samples that differed in terms of cross-linking reaction parameters. Consequently, the degradation rate of the higher and denser cross-linked material is slower, resulting in a final weight loss that is less than that of the material that was cross-linked to a lesser extent.^81^

The utilization of both lignin and cellulose in a single fertilizing composition was described in the work of Li, T. The lignin-based core of the fertilizer was synthesized by mixing of lignin with NH_4_ZnPO_4_ (1:1 wt %) and subsequent granulation with urea particles, thus forming a round, multifunctional fertilizer core. Subsequently, an ethyl acetate solution of CAB and liquid paraffin (LP) was sprayed onto the granules to form the inner layer of the coating. The outer layer was obtained by adding a lignin-based superabsorbent (LBSA) using the alcohol atomization process. The evaluation of delayed nutrients release was conducted through the execution of a soil experiment. The authors compared fertilizers in which the cores were coated with CAB and with dual coating systems based on CAP:LP – LBSA. The results obtained demonstrated that the use of CAB alone was inadequate to meet the SRF criteria. On the first day of the test, the nitrogen released level was found to be above 70%, and the total nutrients released by the granules were fully expended on the 10th day. In contrast, the double coating technology CAP:LP LBSA resulted in approximately 2.5% release on the first day and around 70% release on the 25th day of the test. In addition, a pot experiment was conducted using corn. A primary objective of Li’s research was to synthesize a fertilizer with the capacity to immobilize Pb (II) ions. The pot experiment revealed that the obtained fertilizer exhibited a substantial capacity for Pb (II) ions adsorption and immobilization, thus impeding its migration into the soil environment and subsequent accumulation and absorption by corn during its growth cycle. However, it should be noted that the study did not include a biodegradability investigation. Furthermore, there is a risk that the accumulation of Pb (II) ions over several vegetation periods could be excessive. Nevertheless, the presented approach offers a potential solution for cultivation in contaminated areas.^82^

Based on the data summarized in Table 1, cellulose-based coatings emerge as a promising strategy for EEF formulations, primarily due to their wide availability, low cost, and potential biodegradability of their derivatives. However, a critical examination of the collected studies reveals several directions that require further investigation in future research. A key limitation hindering comparative evaluation of available data is the lack of standardized biodegradation testing protocols. Although the inherent biodegradability of cellulose-derived polymers is frequently cited as a major advantage, the majority of the reviewed studies fail to provide quantitative evaluations of degradation under realistic environmental conditions. Among the references analyzed, only a few^76,79,81^ report data on soil degradation, which are typically limited to short testing periods and lack supporting microbiological or structural analyses. Furthermore, there is substantial variability in coating methodologies and nutrient release evaluation protocols, making direct comparison between studies challenging. Importantly, each coating technique should be considered not only for its efficacy but also for its environmental compatibility and potential for industrial scalability. The reported methods include ethanol-based spray coating and immersion in organic solvents such as acetone, tetrahydrofuran (THF), and chloroform. These solvents pose considerable environmental and operational challenges, particularly on an industrial scale, thus limiting the practical applicability of such formulations. Variations in solvent systems and coating parameters significantly affect film morphology and thickness, which, in turn, are likely to influence nutrient release kinetics and environmental stability. In addition, nutrient release profiles are reported in varying time frames and test conditions, often without including essential environmental parameters such as temperature, soil pH, or soil type. This underscores the urgent need to develop standardized protocols for performance evaluation of coated fertilizers to ensure reproducibility and facilitate meaningful comparison between studies. In terms of release behavior, many cellulose-based coatings exhibit poor initial control, with a substantial portion of nutrients released within the first few hours. For example, a PHB/EC system released more than 80% urea within 30 min under aqueous conditions,^72^ thereby affecting its functionality as a sustained-release matrix. Similar shortcomings have been observed with systems based on cellulose acetate (CA),^72,75^ suggesting inadequate barrier properties under prolonged exposure to water or soil. Of particular note are hybrid formulations incorporating hydrogels, vermiculite or lignin-based superabsorbents, which demonstrate improved modulation of nutrient release over extended periods.^78,79,82^ However, even these advanced systems have seldom been evaluated for their long-term effects on soil physicochemical properties or crop productivity. Only a limited number of studies include^69,77,79^ greenhouse or field evaluations, thus limiting insights into their agronomic performance and practical relevance.

**Table 1:** Examples of the Use of Cellulose in Coated Fertilizers

cellulose modification/non cellulose coatng component	fertilizer	plant growth	method coating	biodegradation test/results	comments test type/nutrient released	ref
ethyl cellulose	urea–clay blend	wheat	spray coating using an ethanol solution	not tested	water test/first 24 h: 37.9%	^69^
					10th day: ∼63%	
ethyl cellulose/starch-SAP	urea	n.a.	spray coating using an ethanol solution	not tested	soil test/1st day: EC, ∼60%; EC-starch, ∼40%	^71^
					4th day: EC-starch, ∼70%	
ethyl cellulose/PHB	urea	n.a.	immersion/spraying using acetone and chloroform solutions	not tested	water test/first 0.5 h: PHB, 90%; PHB-EC, 80%	^72^
ethyl cellulose/P(AA-*co*-AM)	urea	n.a.	immersion using an ethanol solution	not tested	soil test/1st day: 3.5%	^73^
					30th day: 72.8%	
ethyl cellulose	NPK(S)(6-20-30-(7))	n.a.	immersion using an ethanol solution	not tested	water test/1st day: 10%	^74^
					28th day: 70%	
cellulose acetate	NPK(6-20-30)	n.a.	immersion, phase inversion using an acetone solution	not tested	water test/first 5 h: P_2_O_5_, 66.5%; K^+^, 49.6%; NH_4_^+^, 35%	^75^
cellulose acetate/PLA	diammonium phosphate (DAP)	n.a.	spray coating using a THF/acetone (1:1, w/w) solution	4 month soil test: CA 33%; PLA 2.29%	water test/first 24 h: 36%	^76^
					46 h: 100%	
cellulose acetate	urea impregnated biochar	wheat	immersion, phase inversion using an acetone solution	not tested	soil column test/after 10 weeks: 68.15%	^77^
cellulose acetate/ poly(acrylic ac-id-*co*-acrylamide)/unexpanded vermiculite [P(AA-*co*-AM)/UVMT]	NPK(CaMg)	n.a.	immersion, phase inversion using an acetone solution/mechanical sirring	not tested	soil test/3rd day: 4.2%	^78^
					5th day: 8.7%	
					30th day: 72.4%	
cellulose acetate butyrate/CMCS-*g*- PAA/APT	ammonium zinc phosphate	n.a.	spray coating using an ethyl acetate solution	50 day soil	soil test/3rd day: 9.2%	^79^
				soluion test	15th day: 53.1%	
				CA not tested		
				CMCS-*g*- PAA/APT 52.7, 38.8, and 31.5% for N-MCS contents of 1, 2, and 3%	30th day: 81.4%	
hydroxyethylcellulose–carboxymethylcellulose/regenerated cellulose–carboxymethyl cellulose/regenerated cellulose	monoammonium phosphate	n.a.	spray coating	24 day soil test: 63.15%	water test/after the first 1 h: 58%	^81^
					12th h: 100%	
					soil test/1st day: 59%	
					18th day: 100%	
cellulose acetate butyrate: liquid parrafin–lignin-based superabsorbent	lignin: NH_4_ZnPO_4_/urea	corn	spray coating using an ethyl acetate solution	not tested	soil test/1st day: 2.5%	^82^
					25th day: 70%	

### 2.3 Lignin: Structure, Properties, and Industrial Applications

The term “lignin” originates from the Latin word “lignum”, meaning wood. Lignin, which comprises 20–30% of the dry mass of wood, is a naturally occurring polymer that is found primarily in the cell walls of plants. It serves a crucial structural function by providing mechanical strength. Lignin is a vital component of wood, responsible for its hardness and durability, making it the second most abundant natural material after cellulose. Its primary structural role is to maintain the vertical posture of plants and protect cells from decay.^83^

Worldwide, the paper industry produces approximately 50 million tons of lignin annually. Although 95% of industrial lignin is currently used as fuel for heating in boilers, there is a growing market for various products that can be made from lignin. These include adhesives, cement additives that improve curing properties, and as a component of drilling fluids for underwater–oil wells.^84^ The global lignin market generated a revenue of USD 1320.2 million in 2024 and is expected to reach USD 1710.6 million by 2030. The market is expected to grow at a CAGR (2025–2030) of 4.4% by 2030.^85^ The main driving force for this growth is the anticipated increase in demand for lignin in animal feed and natural products. Lignin’s characteristic structure allows for direct utilization, making it an ideal component in the production of macromolecules used in asphalt, biofuels, and catalysts.^86^

Lignin’s hydrophobic nature and neutrality to most solvents and acids make processing it in its native form rare. When a series of chemical processes to modify its physicochemical properties, lignin can be transformed into a highly useful polymer. However, its structure offers ample opportunities for chemical modification, primarily through reactions with the free hydroxyl groups. In particular, lignin can undergo esterification with anhydrides and acid chlorides. Valuable low-molecular-weight products can be easily obtained through depolymerization of lignin. Additionally, lignin-based materials can be obtained by utilizing the similarity of monomeric units to phenol in various resins. This discussion will focus on the functionalization reactions of hydroxyl groups, as lignin chemistry is a broad topic.^87^

Lignin, an amorphous polyphenolic material, is formed by enzymatic polymerization of three phenylpropanoid monomers (monolignols): coniferyl alcohol, sinapyl alcohol, and *p*-coumaryl alcohol in Figure 6. The degradation and proportion of individual monomers depend on the type of plant. Lignin can be categorized according to its origin; for example, lignin from coniferous plants contains the highest amount of coniferyl alcohol units, while lignin from deciduous plants consists of coniferyl and sinapyl alcohol units. The lignin of grasses contains an additional unit of *p*-coumaryl alcohol.^88^ Monolignols are connected by carbon–carbon and ether-type bonds to form a complex three-dimensional network. Unlike cellulose, lignin’s random monomer distribution leads to variations depending on the plant source.

**Figure 6:**
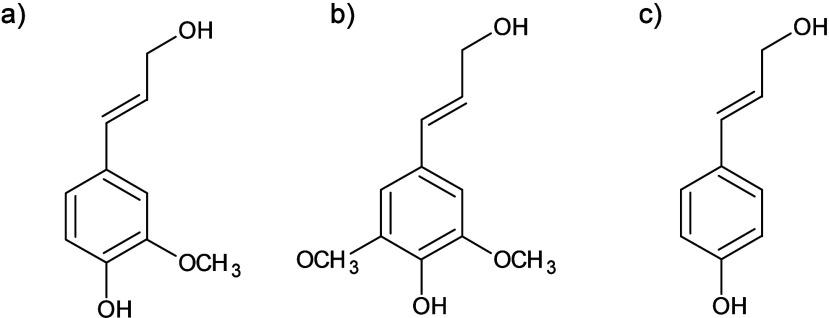
Structural formulas of monolignols: (a) coniferyl alcohol; (b) sinapyl alcohol; (c) *p*-coumaryl alcohol.

Lignin alone as a coating material does not provide sufficient mechanical strength, and its water barrier properties are negatively affected by the presence of hydrophilic phenolic groupings. However, when combined with other coating materials, such as polymeric materials or a multilayer system, lignin can significantly improve its performance.^89^

García et al, demonstrated in their research that a coating layer can be created using polyurethane based on lignin to coat urea. The coating was applied from a solution in a rotary drum using a mixture of lignin and calophony. The study investigated the thickness of the impact of the coating layer on the nitrogen release profile. The article demonstrates the significant impact of an external layer made of linseed oil on the rate of macronutrients. The experiment was conducted under controlled conditions in living organisms using grass (*Lolium perenne*) as control objects. The research unequivocally showed that nitrogen losses are highest in the case of the uncoated fertilizer and decrease with the thickness of the coating applied to the fertilizer. The use of linseed oil as a plasticizer resulted in a decrease in nitrogen losses and an increase in the macronutrients absorbed by the plant without compromising the plasticity of the material. This was achieved with confidence, as indicated by the source.^90^

Lignin, a polymer found in plant waste, can be extracted through technological processes, opening up new opportunities for sustainable use. Its versatile applications include biomaterials, paper production, and serving as a source of biofuels in the energy industry.^91^

Anselme Payen, the scientist who discovered cellulose, is also credited with the discovery of lignin. During the processing of wood with nitric acid and sodium hydroxide, a material with high carbon content was produced, in which cellulose was saturated.^92^

Lignin is a byproduct of various processes that use lignocellulosic biomass as a raw material, such as pulp production or second-generation ethanol. The potential applications of lignin are determined by its type. These properties include composition, molecular weight, molecular structure, solubility, and wettability.^93,94^ Lignin is a byproduct of cellulose fiber separation processes in the paper industry. It undergoes partial degradation during these processes, resulting in a molecular weight than native lignin. The classification of lignin depends on the cellulose separation process used and can be categorized as Kraft, soda, lignosulfonates, or solvent lignin.

Additionally, lignin can replace phenols in existing petrochemical processes. The increasing awareness has led to lignin being considered as a potential natural raw material that can replace petrochemical-derived polymers, thus driving growth in this sector. Continuous product innovations have had a positive impact on the development of lignin-based products. For example, in response to customer needs, the Finnish company StoraEnso, a key lignin producer, introduced granulated lignin.^94^

#### 2.3.1 Chemically Modified Lignin

Lignin is rich in hydroxyl groups that are linked to both the aromatic ring and the linear carbon skeleton. Lignin can be modified through several processes, including esterification, phenolation, etherification, urethanization, and other common organic chemistry reactions that are facilitated by specific groups. Reactions involving hydroxyl groups produce polyol derivatives of lignin, which are more soluble than the raw lignin.^87^ The schematic pathways are shown on Figure 7

**Figure 7:**
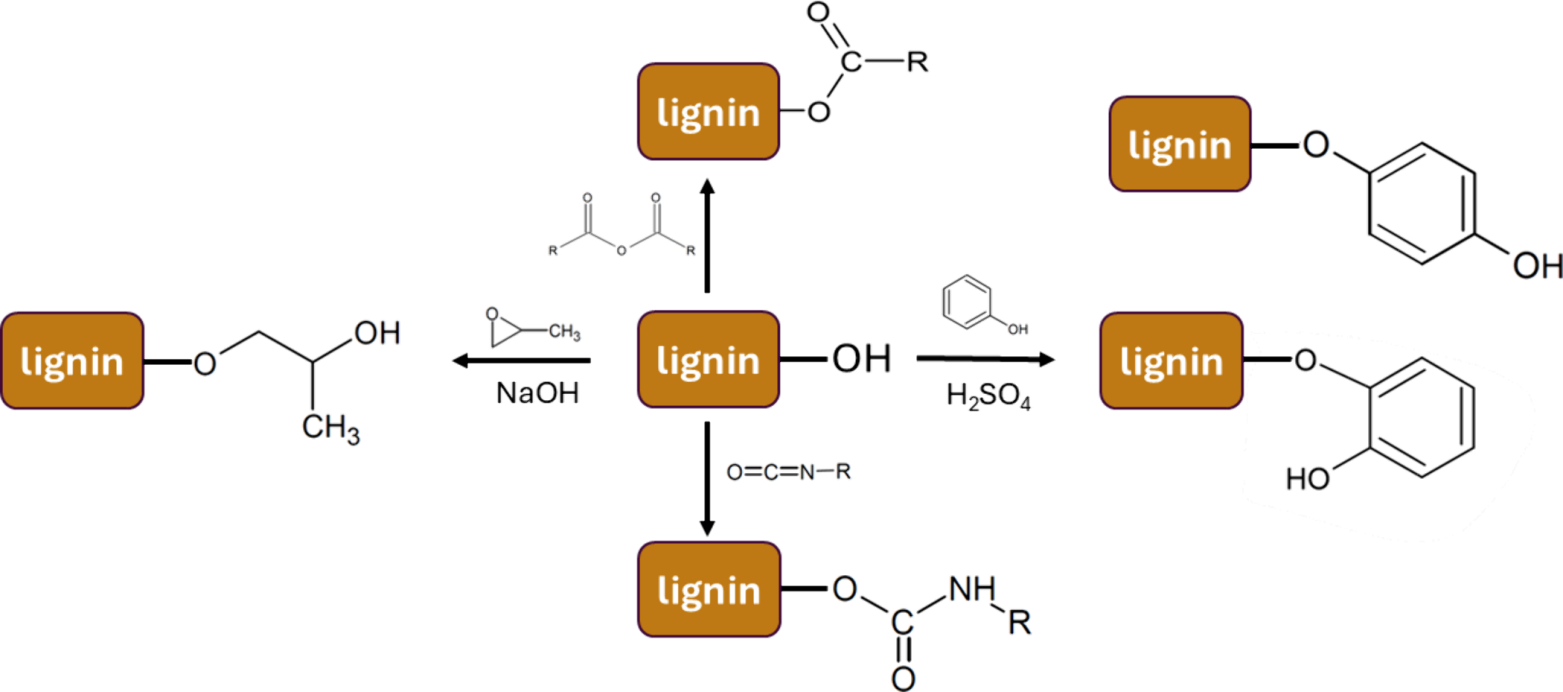
Reaction graph of the possibilities of lignin modification.

Among these processes, esterification appears more frequently in publications. Carboxylic acids and their anhydrides or chlorides are commonly used in these reactions. These processes can be conveniently conducted in a laboratory setting using solvent systems such as dioxane or pyridine. Pyridine serves as both a solvent and a catalyst in the latter case.^95^ Esterification with anhydrides is particularly convenient because it eliminates the need to remove water during the process. When carboxylic acids, water removal during the reaction is necessary to shift the equilibrium toward product formation. Esterified lignin, functionalized through esterification, is a common choice for polymer blends.

Esterified lignin is characterized by superior solubility compared to native lignin. Consequently, it can be utilized in coating methods that employ polymeric solutions. Furthermore, the results of the study demonstrated that modified lignin, through the esterification process, exhibited a decline in glass transition temperature. This finding offers a promising prospect for the synthesis of thermoplastic materials.^96^

Polyol derivatives demonstrate superior compatibility with copolymers such as poly­(3-hydroxybutyrate-*co*-3-hydroxyvalerate) (PHBV) or polypropylene. Liu et al. developed an esterification method that eliminates the main disadvantages of the traditional lignin esterification process. This process involves modifying lignin with ethylene carbonate to produce a derivative containing 90% aliphatic hydroxyl groups, which are subsequently esterified using propionic acid. The excessive use of propionic acid as both a solvent and a reagent in the reaction ensures a highly efficient and effective process.^97^

In contrast to the previous case, the modification of the hydroxyl groups using phenol differs in appearance. Phenolization increases the content of phenolic hydroxyl groups, improving the reactivity of reactions where lignin substitutes for phenol, particularly in the production of biophenolic resins.^98^ This reaction is gaining importance due to the increasing trend of replacing petrochemical raw materials with natural alternatives. In a separate study, cardanol, an alkyl derivative of phenol, was used to substitute phenol. The modified lignin was then utilized to manufacture polyurethanes, which showed increased flexibility and a lower glass transition temperature compared to pure lignin.^99^ The phenolization reaction, where phenol acts as both a reagent and a solvent, is typically conducted at high temperatures and in the presence of sulfuric acid as the catalyst. Modified lignin, with a higher number of phenolic hydroxyl groups, exhibits increased reactivity, making it an excellent substitute for phenol in resin production processes. Although this approach is currently very interesting, it is not applicable to the production of encapsulated fertilizers.^100^

Lignin can be modified through etherification of its hydroxyl groups, with oxypropylation and oxyethylation being the most extensively researched methods.^87^ This method has been well documented and researched, highlighting its effectiveness and reliability. Wu and Liu^101^ described the periodic modification process of lignin through oxypropylation under alkaline conditions using KOH as a catalyst. The reactions were carried out at 180 °C. The presence of lignin in the system almost completely inhibited the competitive reaction, namely, propylene oxide, which occurs at 80 °C in the absence of lignin. The progress of the reaction was monitored by measuring the system pressure drop, which indicates the gas-phase overreaction of propylene oxide. A drawback of the described process is the need for extractive isolation of the resulting polymer, using acetonitrile or hexane. However, there are alternative methods available, such as selective oxypropylation of phenolic hydroxyl groups with compounds like polypropylene glycol, which results in the production of a branched macroscopic molecule with long carbon chains terminated with hydroxyl groups. Lignin is a macroscopic molecule that is frequently utilized in the production of biopolyurethanes, including rigid polyurethane foams.^102^

An effective method of functionalizing lignin is through hydroxyl group reactions, such as urethanization. This process involves the reaction of hydroxyl groups with isocyanate groups of another compound through a urethane bond.

Urethanization offers a sustainable and environmentally friendly alternative to materials obtained using fossil fuel-derived raw materials. The materials obtained have a wide range of applications, similar to classical polyurethanes.^103^ Pretreatment with alkyl oxides is necessary before using lignin to obtain polyurethanes due to its varying structures depending on the source and processing method. Replacing a portion of classical polyol, such as polyethylene glycol, with lignin is a confident strategy.^104^ Similar to the products of phenolization, urethanized lignin is also not used as a coating agent in the production of EEF.

The primary issues are the disposal of waste resulting from reagent use and the use of toxic solvents and catalysts, particularly halogen compounds. Separation of these toxic solvents and catalysts increases costs and environmental impact, making these reactions less appealing to industry. Additionally, longer-chain organic acids are more effective than anhydrides and acid chlorides in modifying lignin. The goal of lignin modification research is to create highly efficient reaction systems that eliminate the need for pyridine and dioxane while reducing waste generation. Such an approach complies with the principles of sustainable chemistry and will lead to significant improvements in this field.^97^

#### 2.3.2 Modified Lignin: EEFs

Lignin’s distinctive properties, such as hydrophobicity, coating-forming ability, and urease-inhibiting characteristics, make it an excellent choice for fertilizer formulations aimed at minimizing nitrogen losses during fertilization. There are three main categories of lignin-containing fertilizers: lignin-coated and derivative fertilizers, chemically modified fertilizers (e.g., with nitrogen introduction), and lignin-based chelate-type fertilizers. Despite the complexity of the structure of lignin, successful modifications can be achieved using various raw materials.

The studies presented by Behin and Sadeghi^105^ exemplify a highly sustainable approach. The starting material, black liquor, is a waste byproduct of the paper industry consisting of dissolved residues of lignin and hemicellulose from pulp, as well as other inorganic chemical compounds used in paper production and processes of extracting cellulose and lignin. This mixture was modified with acetic acid to obtain its acetylated form. The granulated urea was coated with the resulting product in a fluidized bed coating apparatus. Experiments conducted under soil and water conditions demonstrated that coated fertilizers effectively slowed down the release of nutrients. The nutrient release profile was observed to depend on the amount of coating applied. The results presented do not meet current standards for coated fertilizers. However, new recommendations and standards related to the use of biodegradable materials will lead to changes in fertilizer classification studies. The results of the nutrient release do not meet existing standards, and the long-term effectiveness under various conditions remains unassessed. Potential soil or water contamination is not addressed, and the coating’s dependence on precise application lacks clear guidelines. The study’s reliance on anticipated regulatory changes further limits its immediate practicality and relevance. Islam et al.; moreover, they stated that more research is needed to understand the impact of additives on polymer degradation by microorganisms, as numerous reports highlight the harmful effects of microplastics on organisms and soil functions.^106^ Additionally, Gutiérrez et al. emphasize that there is currently no standardized method to determine the biodegradability of the EEF matrix/coating. They underscore the importance of further research on the mechanisms and processes associated with the nutrient release behavior of EEFs, such as soil retention, transport phenomena, and biodegradation processes.^107^ The solution presented, which illustrates the path from waste to a new ecological application, is fully with current trends in sustainable chemistry.

A team from a Swedish laboratory led by Li et al.^108^ investigated and described the use of modified lignin in a polylactide (PLA). The researchers confidently employed softwood kraft lignin modified through acylation with acetic anhydride and palmitic anhydride as coating materials for granulated urea. The modifications were performed using pyridine as the solvent and catalyst. Modified lignin was coated onto urea granules using a mixed PLA solution composed of 60% PLA solution in dichloromethane and a 6% lignin solution in dioxane. The coated granules were dried to obtain the final fertilizer product. The study found that the combination of PLA with lignin modified by acetic anhydride resulted in a pore-free material, unlike the porous structure of PLA. Alternatively, using palmitic anhydride as a modifying agent for lignin results in a nearly smooth material under the microscope. The prepared fertilizers were evaluated through a standard water test. The results showed that the incorporation of palmitic anhydride modified lignin into a PLA blend significantly prolongs the time for nutrient release. Specifically, the study showed that the nutrient release time was extended by a factor of 30 compared to uncoated urea and by a factor of 20 compared to a sample containing only PLA. Although the study demonstrates an extended nutrient release time, it relies on toxic solvents such as pyridine and dichloromethane, raising environmental and safety concerns. Lack of long-term testing under real-world agricultural conditions raises questions about the coating’s durability and effectiveness of the coating over a growing season.

Lignin extracted from Moroccan grass was successfully used as a coating based on material to create a natural resources for fertilizer. The process of preparing the coating involved using a composition of lignin and methylcellulose with citric acid as a cross-linking agent for both polymers. The use of water as a solvent is noteworthy, highlighting the importance of environmentally friendly solutions and workplace safety. The core consists of granulated diammonium phosphate. The coating was evaluated through both a water test and soil analysis, which demonstrated that the use of coatings significantly slows the release of nutrients. Applying a coating of approximately 7.5% results in a 15-fold decrease in the release of both nitrogen and phosphorus. The material obtained showed a high percentage of biodegradation, which improves the ecological value of the fertilizers produced.^109^ Notwithstanding the favorable properties of water as a solvent and the biodegradability of the material, research lacks long-term field tests to verify the coating’s efficacy in various agricultural contexts. The reliance on citric acid as a cross-linking agent raises questions about scalability and cost-effectiveness.

Chen et al.^104^ demonstrated the use of lignin in composite systems for the production of EEFs. In their study, alkali lignin was used as a polyol to obtain polyurethane, which coated the granulated urea. The fertilizers were also coated with paraffin and epoxy resin. The study demonstrates that the fertilizer coated with a composite layer consisting of lignin, wax, and epoxy resin outperformed the fertilizer coated with a monomaterial layer. The evaluation was carried out over a 50-day soil test, using lettuce as a control plant, and the results showed that the composite material released nitrogen more evenly and lasted twice as long compared to the monomaterial layers. These findings provide strong evidence for the effectiveness of the composite coating in improving fertilizer performance. Plants that require consistent and uniform supplementation throughout their growth cycle rely on a crucial release profile. The scalability of the coating process and the cost implications of combining multiple materials remain unexplored. Furthermore, reliance on a single plant species (lettuce) in the 50-day soil test limits the generalizability of the results in diverse crops and growing conditions. The lack of long-term field studies undermines the practical applicability of the findings.

Wei et al.^110^ presented a solvent-free method to coat urea with sodium lignosulfonate that has been cross-linked using epichlorohydrin and then acylated with lauroyl chloride. The modified lignin, with a glass transition temperature of around 90 °C, was finely ground and mixed with preheated urea at 100 °C. This process caused the urea to melt and effectively coat the fertilizer granules. The process was repeated until the desired degree of coverage was achieved. The coated fertilizer was then evaluated in a soil test without the use of plants. Modified lignin-coated urea, comprising approximately 62% by weight, released approximately 88% of nutrients on day 44, as evaluated following the Chinese standard No. T23348-2009 for SRFs. The team’s solvent-free method for coating fertilizers is noteworthy for being environmentally friendly. However, using such a high mass ratio of coating to fertilizer is economically inefficient and impractical for large-scale implementation. However, the study has significant limitations. The use of a high coating-to-fertilizer ratio (62% by weight) is economically inefficient and impractical for large-scale agricultural use, limiting its feasibility. Additionally, the evaluation lacked plant-based soil tests, which are critical to assessing agronomic performance under real-world conditions.

Sadeghi’s publication^111^ presented a comparison between a fertilizer coated with an innovative lignin material and a commercially available sulfur-coated fertilizer. The experiment used waste lignosulfonate, which was modified by acylating it with oxalic acid in a water environment. In the experiment carried out under soil conditions, the resulting material was applied using a fluidized bed coating apparatus. The experiment compared urea-coated fertilizers with modified lignin and sulfur. The results of the soil test showed that on the 18th day of the trial, the commercial fertilizer released 93% of nutrients, while the fertilizer with the author’s material released 79%. In addition, the publication highlights attempts to synthesize modified lignin using other organic acids. The study unequivocally demonstrates that acetic and adipic acids react under the given reaction conditions to form a water-soluble product, unlike oleic and stearic acids. The use of stearic acid in the reaction aligns perfectly with the principles of green chemistry by promoting the use of water as a solvent. The environmental and economic feasibility of scaling the fluidized bed coating process and the use of oxalic acid as a modifying agent remains unclear. Furthermore, the study lacks long-term soil tests to evaluate the durability and performance over an entire growing season. Although the use of stearic acid aligns with the principles of green chemistry, the findings on other organic acids offer limited practical guidance for optimizing the process.

Fertahi et al.^112^ describe the use of lignin extracted from olive pomace, blended with carrageenan, a naturally derived polysaccharide obtained from red algae and commonly used in food products and jellies. The lignin was sourced from waste olive pomace and then plasticized with PEG 200. The resulting material was applied to the granulated phosphorus fertilizer in a fluidized bed coating drum. Coated fertilizers containing lignin, carrageenan, and their composites, both with and without the addition of PEG 200, were prepared and evaluated in a standard water test. The test determined the amount of phosphorus released, and on the fifth day, each fertilizer showed 100% phosphorus release. Upon observation of the release profile, it was determined that the fertilizer with the lignin/carrageenan compound released approximately six times less phosphorus than the uncoated fertilizer after the first day of the test. The addition of PEG 200 did not affect the phosphorus release profile, but improved the mechanical properties of the material. The fertilizers released 100% of their phosphorus by the fifth day, failing to meet the criteria for SRFs. Additionally, while PEG 200 improved mechanical properties, its lack of impact on nutrient release limits its practical value. The absence of long-term soil or plant-based tests further undermines the study’s ability to assess the coating’s real-world performance and environmental sustainability.

Another study also described a phosphorus fertilizer based on modified lignin. Rotondo et al.^113^ outline the process of obtaining fertilizers coated with lignin modified in various ways. The lignin utilized in the Rotondo work was part of eucalyptus kraft black liquor. The first modification entails a mixture of phenol-formaldehyde resin and hydroxymethyl lignin, with talc added as a binding agent. The second modification involves a mixture of acylated lignin, acetic anhydride, and CA. The fertilizers were evaluated and compared in a water test. A noteworthy sample contained 23.5% by weight of modified lignin of the first type, which did not release more than 15% of the nutrients after the first day. Therefore, further testing is required before conclusions can be made. It is important to note that the test only lasted 3 days, which is not enough time to determine whether the fertilizer would meet all the release requirements. As a result, the remaining samples were rejected. Based on preliminary selection and the promising values of the released substance at the end of the test (18% released phosphorus), it is reasonable to reject the remaining trials.

The utilization of lignin-based polyurethane for coating granulated urea was the subject of investigation by Chen X.^114^ The synthesis of lignin-based polyurethane materials was achieved through the modification of alkali lignin with PEG 400, poly­(ε-caprolactone) diol (PCLD), and glycerol under acidic conditions. Subsequently, the liquified lignin (LL) was mixed with polymethylene poly­(phenylene isocyanate) (PAPI) to obtain a coating solution. The granulated urea was then coated in a rotary drum with the liquid mixture of coating materials, which cured in the coating process at the evaluated temperature (70 °C) in 6 min. Additionally, the composition of the coating mixture was enhanced with carbon black and polysiloxane, which were prepared for the same purpose. The final stage of the process involved the preparation of three different EEF compositions: lignin-based polyurethane coated urea (LPCU), carbon-lignin-based polyurethane-coated urea (CLPCU), and hydrophobic lignin-based polyurethane-coated urea (HLPCU). The nutrient release performance of the fertilizers was measured at room temperature in water for 49 days. In addition, a 60-day test was performed using cabbage, with HLPCU fertilizer. The authors concluded that the rate of nitrogen release from HLPCUs was significantly slower than that from LPCUs and CLPCUs. In experiment three, various percentages of coating were applied: 3, 5 and 7%. It was found that the rate of nitrogen release decreased as the amount of coating increased. Samples with 7% coating and varying levels of polysiloxane were examined. The results indicated that a 20 wt % of polysiloxane yielded 2% after 1 day, 10% after 7 days, and 86% at the end of the test. The objective of the pot trials was to verify the growth-promoting effect of HLPCU on cabbage plants. The objective of the investigation was to make a comparison between HLPCU and a traditional urea treatment, as well as a control (CK), to assess plant growth, nitrogen (N) use, and soil nutrient dynamics. The degradation test in a soil environment demonstrated that after 60 days, 120 days and 180 days, the mass loss was 9.16%, 19.13%, and 33.40%, respectively. The results obtained from the degradation test in a soil environment provide evidence to support the hypothesis that the material exhibits biodegradability properties.

Boonying’s work^115^ were described the utilization of nonmodified lignin for the coating of granular urea fertilizer. By combining kraft lignin with prevulcanized natural rubber latex (pVLA) and additionally adding beeswax and spraying this composition on urea, we obtained the EEF (w-Lignin/pVLA-CRF). The study evaluated four types of coated fertilizers: lignin, pVLA, lignin/pVLA and bee wax-lignin/pVLA coated in a pancoating machine. The evaluation studies were conducted in both water and soil conditions. Results of the nutrient released according to ISO 21263:2017: *Slow-Release Fertilizers-Determination of the Release Method of Nutrients for Coated Fertilizers*. These allow one to classify fertilizers as slow-releasing. Uncoated urea shows 100% dissolution in less than 5 min. Samples that were coated only with pVLA, lignin or bees were completely released after about 1 h of test but in contrast to w-Lignin/pVLA-CRF released 86% in 224 days. When subjected to soil conditions, the uncoated fertilizer demonstrated 100% nitrogen release in a time period of less than 7 days, while w-Lignin/pVLA-CRF released nutrients over a period of 7 months. To provide a sustainable approach, biodegradation tests were performed according to ISO 14855–1:2012 to determine the ultimate aerobic biodegradability of plastic materials under controlled composting conditions. The findings indicate that the lignin/pVlA composite material during the 77 days of the experiment was 31.5% biodegradable, compared to 54.6% for pure pVLA. It is reasonable to conclude that these values are satisfactory and allow for the assumption that the material will not accumulate in the soil. The pot test was not included in the scope of this study.

In the recent work by Bouchtaoui,^116^ lignin nanoparticles (LGe-NPs) were successfully incorporated into carboxymethylcellulose (CMC) and used to coat granular DAP. The lignin used in this study was isolated from natural Alfa fibers (*Stipa tenacissima* L.). The composition of the LGe-NP/CMC casting mixture was then sprayed onto DAP granular fertilizer in a coating machine, where it was distributed uniformly. Leaching experiments were conducted to study the release of nutrients in water conditions. As the level of lignin increased, there was a concomitant decrease in nutrients release, reaching 12.08% for P and 5% for N after the first hour of the test. The maximum amount of release for CMC60/LGe-NPs40 occurred after 51 h for phosphorus and approximately 55 h for nitrogen. Furthermore, the findings of the nutrient leaching test conducted under soil conditions were presented. The investigation revealed that the same coated fertilizer released 3.10% of P and 6.88% of N. At the conclusion of the 100-day leaching period, the coated fertilizer had released up to 27.02% of P and 25.06% of N. The study investigated the biodegradability of the films in soil over a 30-day period by monitoring their weight loss. The unmodified CMC film exhibited substantial degradation, with a weight reduction in weight. On the contrary, the incorporation of increasing concentrations of LGe-NP resulted in a progressive decline in degradation rates, with values of 11.20% for CMC60/LGe-NPs40. This reduction in degradability was attributed to the enhanced hydrophobicity of the nanocomposite films, which impedes water penetration and consequently favors slower surface erosion over the more rapid bulk degradation observed in hydrophilic materials. In order to carry out a complete characterization of the prepared fertilizers, pot tests were performed. The subject of the investigation was the influence of coated DAP fertilizers on wheat development, yield performance, and grain quality. The results obtained from this study indicate that CMC60/LGe-NPs40 has a significant effect on wheat productivity and grain quality, while simultaneously improving nutrient use.

As demonstrated in Table 2, there is increasing interest in lignin as a component in biobased fertilizer coatings. The versatility of lignin in advanced material design is demonstrated by various chemical modifications, including esterification and blending with biodegradable polymers such as PLA and CMC. Notwithstanding this progress, several critical knowledge gaps remain. One such aspect pertains to the broad spectrum of coating methodologies employed, encompassing solvent-based spray coating (e.g., ethanol, dichloromethane, dioxane), dip-coating, and thermally assisted mechanical application. Although these techniques are effective at the laboratory scale, there are concerns regarding their industrial scalability and environmental safety. The utilization of volatile organic solvents, in particular, poses significant challenges related to toxicity and emissions, which may impede their practical application and commercial viability. A significant limitation observed across the studies is the absence of biodegradation assessments conducted under realistic environmental conditions. In many cases, the degradation behavior of the substances is not tested or only evaluated in aqueous media, which may not reflect the complexity of soil environments. Only a limited number of references 104,114–116 provide soil degradation data, yet even these lack the detailed microbiological or physicochemical analysis necessary to understand degradation mechanisms. Nutrient release profiles vary significantly, ranging from rapid release (up to 80% within a few days) to more sustained release behavior over extended periods. Hybrid systems that combine lignin with hydrophilic additives such as κ-carrageenan, polyethylene glycol or lignin-based nanoparticles (LGe-NP) are particularly promising. These hybrid systems have been shown to offer improved control over nutrient release, suggesting the potential for precise modulation of nutrient availability. However, most studies remain limited to laboratory-scale evaluations under controlled conditions. Additionally, the scarcity of crop response data, for example lettuce or wheat, hinders understanding the agronomic effectiveness of the treatments. Furthermore, the omission of critical environmental variables, such as soil pH, moisture, temperature, and soil type, hinders greater applicability and interpretation of the results in agricultural systems. In conclusion, while lignin and its derivatives have strong potential as ecofriendly coating materials for CRFs, more work is needed to establish standardized testing protocols. Integration of biodegradation studies, structural characterization, and long-term evaluations of soil and plant performance is recommended for future research. Only through such a comprehensive approach can the practical relevance and environmental compatibility of lignin-based fertilizer coatings be reliably assessed.

**Table 2:** Examples of Utilizing Lignin in Coated Fertilizers

lignin modification/non-lignin coating component	fertilizer	plant growth	method coating	biodegradation test/results	comments test type/nutrient released	ref
alkai lignin/paraffin	urea	lettuce	spray coating	180 day soil test: 23.6%	water test/lignin-coated:	^104^
					3rd day: 36%	
					28th day: 100%	
					lignin/paraffin-coated:	
					3rd day – 24%	
					28th day – 98%	
acetylated sulfite lignin	urea	n.a.	spray coating using an ethanol/dichlormethane solution	not tested	soil test/4th day: 30%	^105^
					12th day: 60%	
					20th day: 82%	
acetylated kraft lignin	urea	n.a.	spray coating using an ethanol/dichlormethane solution	not tested	soil test/4th day: 22%	^105^
					12th day: 45%	
					20th day: 68%	
acetylated kraft lignin/PLA	urea	n.a.	dip-coating process using a dichloromethane solution for PLA and a dioxane solution for lignin modificaton	not tested	water test/1st day: 3.5%	^108^
					30th day: 72.8%	
palmitynated kraft lignin/PLA	urea	n.a.	dip-coating process using a dichloromethane solution for PLA and a dioxane solution for lignin modification	not tested	water test/1st day -10%	^108^
					28th day: 70%	
esterified cross-linked sodium lignosulfonate	urea		mechanical sticking under elevated temperature	not tested	soil test/44th day: 86%	^110^
modified lignin sulfonate with oxalic acid	urea		spray coating in fluidized bed using ethanol solution	not tested	water test/after 45 h: 100%	^111^
lignin/κ-carrageenan; PEG 200	triple superphosphate (TSP)		spray coating	not tested	water test/after 6 h: 13.51%	^112^
					3rd day: 70.46%	
lignin-based polyurethane/polysiloxane	urea	cabbage	spray coating	soil test/60th day: 9.16%	water test/after 24 h: 1.24%	^114^
				120th day: 19.13%		
				180th day: 33.40%	60th day: 85%	
w-Lignin/pVLA	urea	n.a.	spray coating	soil test/after the 77th day	water test/224th day: 86%	^115^
				pVLA: 53.6%		
				lignin/pVLA: 31.5%	soil test/7th month: 100%	
lignin nanoparticles/CMC	DAP	wheat	spray coating	soil test/after the 30th day: CMC 60.50%; CMC60/LGe-NPs40, 11.20%	water test/after the first 1 h: P, 12.08%; N, 5%	^116^
					after ∼50 h: P and N, 100%	
					soil test/after the 30th day: P, 3.10%; N, 6.88%	
					after the 100th day: P, 27.02%; N, 25.06%	

### 2.4 Potential of Cellulose and Lignin in Coated Fertilizers

The utilization of natural polymers, such as cellulose and lignin, in the production of coated fertilizers shows promising potential in agriculture and environmental protection. Research suggests that the use of cellulose and lignin as fertilizer coatings can provide several benefits. The application of cellulose and lignin-based coatings facilitates the controlled release of nutrients, allowing the fertilization to be better adapted to the needs of plants at different stages of their growth. This controlled release mechanism synchronizes nutrient availability with plant uptake, enhancing NUE and reducing the frequency of fertilizer applications. For instance, CA coatings have been shown to significantly slow down the release of nitrogen, phosphorus, and potassium, thereby minimizing nutrient losses and environmental pollution. Fertilizers coated with natural polymers have been shown to reduce nutrient losses through leaching and volatilization. This results in improved nutrient uptake by plants and increased overall fertilizer efficiency. Studies have demonstrated that both lignin- and cellulose-based EEFs can extend the release time of nutrients from a few days to several weeks, thereby reducing the need for frequent applications. The use of natural polymers can also improve the structure of the soil by increasing its ability to hold water and nutrients. Consequently, this renders the soil more fertile and conducive to plant growth. Furthermore, fertilizers coated with natural polymers can reduce soil erosion, which is important for maintaining soil fertility and protecting against degradation. Cellulose and lignin-based coatings have been shown to enhance soil aggregation and water retention, thus promoting optimal root development and increased crop yields.

The utilization of biodegradable materials such as cellulose and lignin is in accordance with the principles of sustainable agriculture. These materials are subject to natural decomposition in the soil, thereby reducing the accumulation of nonbiodegradable polymers and microplastics. This contributes to the mitigation of adverse effects associated with conventional fertilizers and supports the development of more sustainable soil management practices. The combination of cellulose and lignin with other materials to create multilayer compositions has the potential to further enhance their performance. According to the European Union Regulation on fertilizing products, it is mandatory that all fertilizer components and their packaging be made of biodegradable materials by July 2024. This regulatory shift underscores the importance of developing fertilizers with biodegradable coatings. Cellulose and lignin-based coatings meet these requirements and offer a viable solution for compliance with new regulations. The inherent biodegradability of these materials is pivotal in preventing long-term soil contamination and maintaining soil health.

To maximize the potential of natural polymers in the production of coated fertilizers, further research is required to optimize production processes and evaluate the impact of these fertilizers on the environment and plant health. The development of standardized methods for the evaluation of the biodegradability and nutrient release profiles of these coatings is of critical importance. Furthermore, research should focus on the long-term effects of these coatings on soil properties, microbial activity, and crop productivity.

The adoption of cellulose- and lignin-based coatings in agriculture also presents economic benefits. These natural polymers are abundant and cost-effective compared to synthetic alternatives. The utilization of lignin from the paper industry and cellulose from agricultural residues not only reduces production costs, but also fosters circular economy practices. Practical considerations include the scalability of coating processes and the development of efficient application methods to ensure uniform coating and optimal performance.

## 3 Challenges, Opportunities, and Outlook

This comprehensive review of biodegradable cellulose- and lignin-based coatings for EEFs demonstrates the significant potential of these biopolymers in sustainable agricultural applications. A systematic analysis of the literature reveals a clear progression in coating design methodologies, moving from elementary single-layer systems to sophisticated composite and multilayer architectures. A critical evaluation of existing coating techniques indicates that various application methods have been studied, with growing emphasis on reducing environmental impact from organic solvents. However, widespread implementation of these systems remains limited by the lack of standardized protocols for biodegradation assessment and insufficient data from longitudinal field experiments. A particularly promising research direction is the adaptation of nutrient release from fertilizer granules to the specific physiological requirements of different crop species at all stages of their development. Modification of the physicochemical properties through selective functionalization of cellulose and lignin facilitates synchronization between nutrient bioavailability and plant growth stages, potentially increasing fertilizer use efficiency. Experimental studies suggest that optimized coating formulations can significantly improve nitrogen use efficiency compared to conventional fertilizer systems, potentially reducing nitrogen losses in agriculture systems. In order to successfully commercialize these materials, it is necessary to develop scalable production methods that maintain economic viability while preserving precise control over biodegradation mechanisms and nutrient release profiles. Future research directions should include comprehensive life-cycle assessments and detailed studies of the interactions between biodegradable coatings and soil microorganisms. Such integrated approaches will facilitate the development of next-generation fertilizer systems that simultaneously meet agricultural productivity requirements and environmental sustainability goals.

## Supplementary Material



## Notes

Systematic review flow diagram: the PRISMA flow diagram for the systematic review detailing the database searches, the number of abstracts screened, and the full texts retrieved (PDF)

## Abbreviations

CA: cellulose acetate
CAB: cellulose acetate butyrate
CAGR: compound annual growth rate
CRF: controlled-release fertilizer
DS: degree of substitution
EC: ethyl cellulose
EEF: enhanced-efficiency fertilizer
PLA: polylactide
SRF: slow-release fertilizer
