# Supplementary material for: Sustainable Agriculture Solutions: Biodegradable Coatings for Enhanced-Efficiency Fertilizers Using Cellulose and Lignin
Source: J Agric Food Chem. 2025 May 20;73(22):13105–24. doi: 10.1021/acs.jafc.5c01173 (PMC12147134; doi:10.1021/acs.jafc.5c01173)
Supplement: Supplementary file 1 [file jf5c01173_si_001.pdf]

Supporting Information

Sustainable Agriculture Solutions: Biodegradable Coatings for Enhanced-efficiency fertilizers (EEF). Using Cellulose and Lignin

Przemysław Boberski[1,2,*], Marek Główka[1,2] , Kamila Torchała[1], Grzegorz Kulczycki[3] and Nikodem Kuźnik[2]

[1] Łukasiewicz Research Network – Institute of Heavy Organic Synthesis “Blachowania”, ul. Energetyków 9, 47-225 Kędzierzyn-Koźle, Poland

[2] Faculty of Chemistry, Silesian University of Technology, ul. M. Strzody 9, 44-100 Gliwice, Poland

[3] Institute of Soil Science, Plant Nutrition and Environmental Protection, Wroclaw University of Life and Environmental Sciences, ul. Grunwaldzka 53 street, 50-375 Wroclaw, Poland

Correspondence:

przemyslaw.boberski@icso.lukasiewicz.gov.pl; tel.: +48 507 940 570

PRISMA 2020 flow diagram for new systematic reviews which included searches of databases and registers only

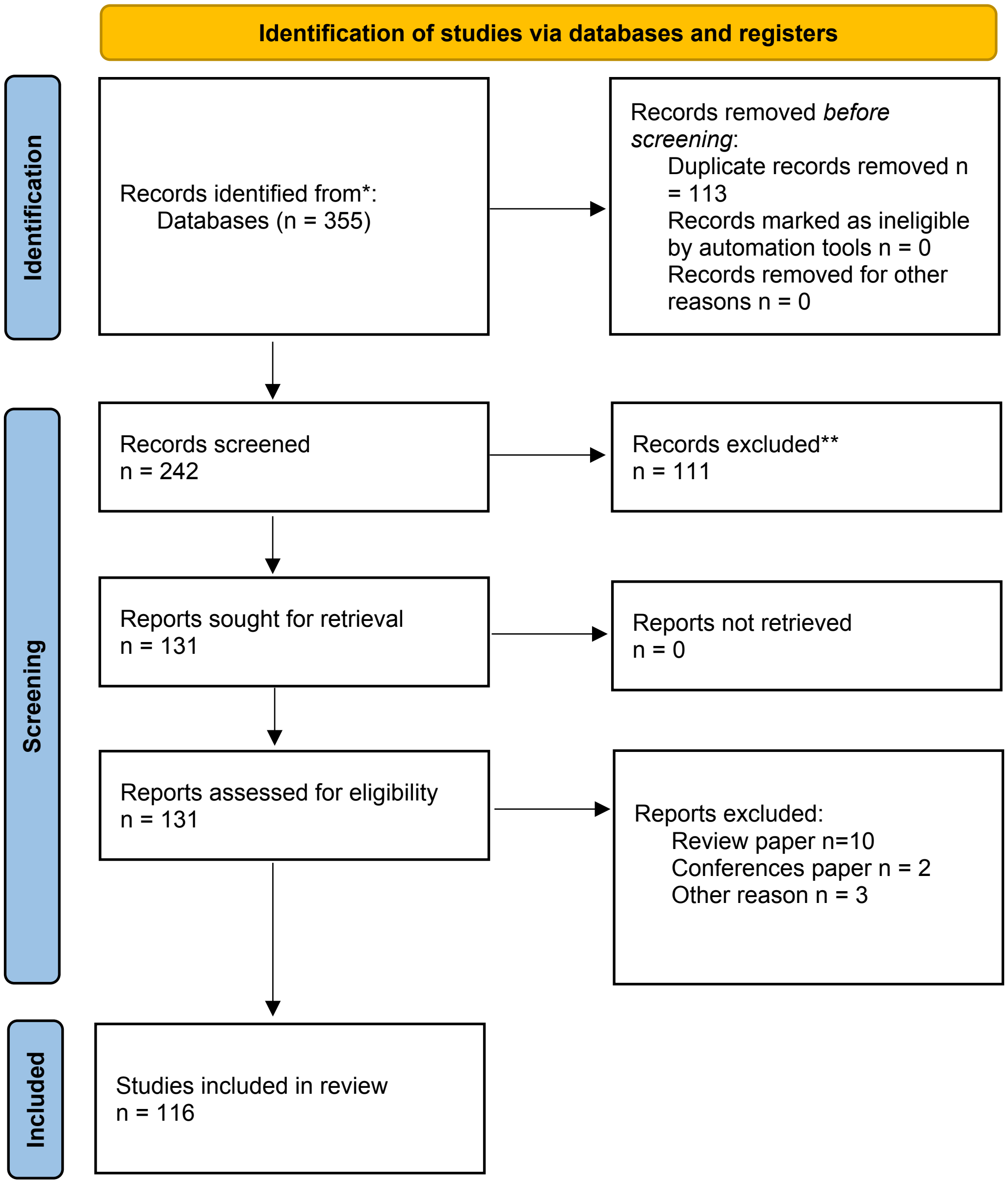

Source: Page MJ, et al. BMJ 2021;372:n71. doi: 10.1136/bmj.n71.

This work is licensed under CC BY 4.0. To view a copy of this license, visit https://creativecommons.org/licenses/by/4.0/
